# The urban morphology on our planet – Global perspectives from space

**DOI:** 10.1016/j.rse.2021.112794

**Published:** 2022-02

**Authors:** Xiao Xiang Zhu, Chunping Qiu, Jingliang Hu, Yilei Shi, Yuanyuan Wang, Michael Schmitt, Hannes Taubenböck

**Affiliations:** a Department of Aerospace and Geodesy, Data Science in Earth Observation, Technical University of Munich, Arcisstraße 21, Munich 80333, Germany; b Remote Sensing Technology Institute, German Aerospace Center, Münchener Straße 20, Weßling 82234, Germany; c Department of Aerospace and Geodesy, Chair of Remote Sensing Technology, Technical University of Munich, Arcisstraße 21, Munich 80333, Germany; d Remote Sensing Data Center, German Aerospace Center, Münchener Straße 20, Weßling 82234, Germany; e Institute for Geography and Geology, Julius-Maximilians-Universität Würzburg, Würzburg, Germany

**Keywords:** Remote sensing, Sentinels, Big data, Data fusion, Deep learning, Local climate zones, Urban morphology, Global urban LCZ dataset, Global inequality

## Abstract

Urbanization is the second largest mega-trend right after climate change. Accurate measurements of urban morphological and demographic figures are at the core of many international endeavors to address issues of urbanization, such as the United Nations’ call for “Sustainable Cities and Communities”. In many countries – particularly developing countries –, however, this database does not yet exist. Here, we demonstrate a novel deep learning and big data analytics approach to fuse freely available global radar and multi-spectral satellite data, acquired by the Sentinel-1 and Sentinel-2 satellites. Via this approach, we created the first-ever global and quality controlled urban local climate zones classification covering all cities across the globe with a population greater than 300,000 and made it available to the community (https://doi.org/10.14459/2021mp1633461). Statistical analysis of the data quantifies a global inequality problem: approximately 40% of the area defined as compact or light/large low-rise accommodates about 60% of the total population, whereas approximately 30% of the area defined as sparsely built accommodates only about 10% of the total population. Beyond, patterns of urban morphology were discovered from the global classification map, confirming a morphologic relationship to the geographical region and related cultural heritage. We expect the open access of our dataset to encourage research on the global change process of urbanization, as a multidisciplinary crowd of researchers will use this baseline for spatial perspective in their work. In addition, it can serve as a unique dataset for stakeholders such as the United Nations to improve their spatial assessments of urbanization.

## Highlights

• Combined big and multisensory Earth observation data with deep learning.
• Revealed the first time detailed morphology of urban agglomerations across the globe.
• Statistical study of our results quantifies a global inequality in population density.
• Clustering of the global result identified seven unique urban morphological patterns.
• This global urban morphological dataset (So2Sat Global Urban LCZ) will be open access.

## 1 Introduction

### 1.1 Motivation

Urbanization is one of the most important trends in global change. Although a near-perfect correlation is verified between urbanization and economic prosperity of societies (Glaeser et al., 2013), this correlation does not automatically lead to a golden future: instead, the unprecedented dynamics and dimensions of natural growth of and migration into cities pose fundamental challenges to our human societies across the globe. Many international endeavors addressing issues of urbanization, such as the United Nations’ call for “Sustainable Cities and Communities,” are based on accurate measurements of urban morphological and demographic figures. Such measurements provide key scientific foundations for the allocation of valuable resources for a wide range of stakeholders and form the basis of global efforts to understand and track progress in improving human livelihoods.

Although urban geographic information has significantly improved in recent decades, the required geo-information is still not available in many countries. For example, the United Nations population figures, on which our understanding of urbanization at these scales is currently primarily based, do not provide information on the distribution, pattern, and evolution of the built environment (Zhang and Seto, 2011). Remarkably, in 2014, for the first time, the IPCC report included a specific chapter on urban areas, making note that cities of sufficient density and spatial scale can influence their local micro-climate (Revi et al., 2014). This highlights the importance of cities to climate action, yet there are significant gaps in knowledge and strategic directions. Global information on inter- and intra- urban variability, urban dynamics, 2D and 3D urban form, and links between urbanization and well-being are overdue (Chai and Seto, 2019, Mahtta et al., 2019). In Arellano Ramos and Roca Cladera (2016), the data of nighttime lights was utilized for detecting and classifying urban areas. Recently, the global land cover map (GLC), which includes urban and non-urban classes, is transferred from 30 m to 10 m (Gong et al., 2019).

In the past decades, satellite remote sensing has been the foundation of data collection and the development of knowledge about our Earth. During that period, new initiatives in global urban mapping have advanced the quality of spatial knowledge. On a global scale, there is a handful of global urban land use classification maps. For instance, the Global Urban Footprint (GUF) (Esch et al., 2013) provides a binary mask of urban vs. non-urban surfaces, while the World Settlement Footprint Evolution (WSF-Evo) (Marconcini et al., 2020) documents their evolution over time. In addition, the World Urban Database provides intra-urban local climate zone classification maps (Stewart et al., 2014, Stewart and Oke, 2012) – however only for about 100 metropolises and to a best resolution of a few hundred meters. Despite their significant contribution to large scale urban mapping, these approaches have not addressed the variations of intra-urban morphology. On the other hand, the potential of Earth observation to describe the complex, small-scale morphology of cities has been shown in many approaches, e.g. (Taubenböck et al., 2013, Bechtel et al., 2015a). However, global urban mapping approaches still lag behind. Consistent and resilient global spatial data in high geometric, thematic and temporal resolutions necessary to address the challenges described above is still nonexistent. This is mainly due to the lack of high resolution global satellite data and computationally and methodologically efficient algorithms.

In the meantime, Earth observation (EO) has irreversibly arrived at a golden era of big data. The game changer is the Sentinel satellites of the European Copernicus programme which provide continuous, reliable, quality controlled acquisition of big EO data that are free and open. To date, tens of petabytes of satellite data from complementary sensors have already been acquired and shared. In addition, the programme offers a long-term perspective of guaranteed data acquisition and sharing until 2030. Recently, the European Space Agency (ESA) awarded contracts for the development of six new Copernicus missions. Beyond that, plans for 2040 are already under discussion. To effectively retrieve global urban geo-information from such a massive data source requires not only new technological approaches to manage large amounts of data, but also new analysis methods. Here, methods of data science and artificial intelligence (AI), such as machine learning, become indispensable. Deep learning in particular has led to a revolution in AI in recent years. Since taking off several years ago, deep learning in remote sensing has become a blooming research field (Zhu et al., 2017, Tuia et al., 2021, Camps-Valls et al., 2021). Its huge potential in global urban mapping using EO data is ripe for discovery.

### 1.2 Review of local climate zones classification algorithms

Local climate zones (LCZs) were originally developed for metadata communication of observational urban heat island studies (Stewart, 2011). But soon they also showed potential in urban morphology mapping. A significant part of the existing development of LCZ classification is community-based large-scale LCZ mapping using freely available Landsat data and softwares (Mills et al., 2015, Bechtel et al., 2015a, Bechtel et al., 2015b, Hidalgo et al., 2019), known as the World Urban Database and Portal (WUDAPT). These community-based efforts mark the first step towards a more synergetic cooperation among researchers. Yet, (Bechtel et al., 2017, Bechtel et al., 2019) discovered that the quality of these maps is heavily dependent on individual producers. (Ren et al., 2016, Ren et al., 2019) also argue that the standard WUDAPT mapping approach cannot fulfill quality demands of practical usage. Therefore, researchers also attempt in parallel to improve the classification methodologies. In terms of data sources, Bechtel et al. (2016) and Hu et al. (2018) demonstrate that synthetic aperture radar (SAR) data can be beneficial as an additional data source. Unger et al. (2014), Zheng et al. (2018) and Zhang et al. (2019) conclude that geographic information system (GIS) data are also beneficial to this classification, subject to the availability of GIS data. Regarding the classification algorithms, similar to WUDAPT, many studies utilized Random Forests to accomplish LCZ classification (Danylo et al., 2016, Demuzere et al., 2019, dos Anjos et al., 2017, Yokoya et al., 2017) due to its easy implementation and robust performance. However, Yoo et al. (2019) and Rosentreter et al. (2020) recently found that convolutional neural networks (CNNs) outperform Random Forests on LCZ classification with fair evidences. Sukhanov et al. (2017) fuse the predictions of random forest- and CNN-based classifiers to deliver LCZ maps. Qiu et al. (2019) propose a recurrent network to utilize multi-temporal Sentinel-2 data for LCZ classification of European cities. Qiu et al. (2020) report the performance of many standard CNN architectures for LCZ classification in a practical and comprehensive manner. The authors also introduced a CNN which utilizes multi-scale representations.

### 1.3 Contribution of this paper

Here we demonstrate a novel deep learning and big data analytics approach in order to fuse freely available global radar and multi-spectral satellite data, acquired by the Sentinel-1 and Sentinel-2 satellites. Via this approach, we achieved the first-ever global and quality controlled urban LCZs classification covering all cities with a population greater than 300,000, i.e., 1692 cities in total, and made it available to the community. We validated our approach on 52 urban agglomerations across all five inhabited continents. To do so, we used different strategies, giving upper and lower bounds to the accuracy of our global classification result. This dataset, named “*So2Sat Global Urban LCZ* (So2Sat GUL^2^),” will boost research on the global change process of urbanization, as a multidisciplinary group of researchers may use this baseline for the spatial perspective of their work - including planners, demographers, sociologists, economists, climatologists, and many others. On top of that, *So2Sat GUL* can serve as a unique dataset for stakeholders such as the United Nations to improve their spatial assessments of urbanization.

To stimulate the dataset's usage, we carried out a study to showcase urban geographic analysis to better quantify and understand the global urban morphology. We found that more than 30% of the human settlement area is sparsely built. Similarly, about 25% of human settlements are lightweight or large low-rise buildings, which are often slum areas in cities of the Global South. By combining the urban morphological map with a global population map, we discovered that the 30% sparsely built area contains less than 10% of the total population of the 1692 cities, whereas the 25% low-rise area contains 26% of the total population. In addition, we were able to show that the influence of geographical and cultural regions on the physical design of the urban landscape also empirically stands up to observations and theories. This quantifies the very uneven distribution of population and settlement types across the world.

The remainder of this paper is organized as follows. The next section briefly introduces the study area and data. Section 3 explains our method for the mapping of global local climate zones. Section 4 presents the experimental results. Section 5 showcases a pilot application of the resulting LCZ map by analysing the relation between LCZ patterns and urban morphology. Finally, in Section 6 we share our perspectives on possible usage of our results for the analysis of global urbanization and on further improvements on our results.

## 2 Study area and data

### 2.1 Study area

**Fig. 1:**
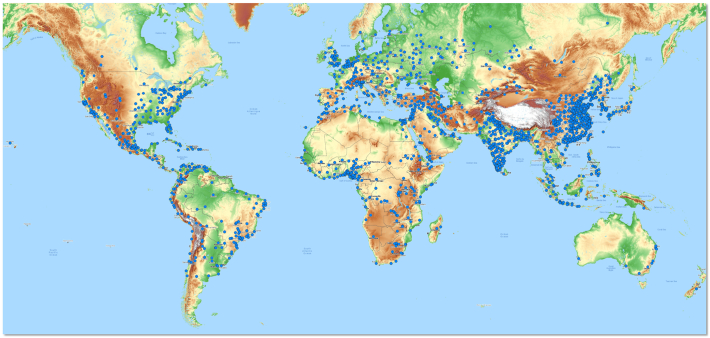
Location of the cities included in this study. The blue dots on the upper figure indicate the location of each city. The background image shows the topographic height of the Earth surface (source: OpenTopoMap (CC-BY-SA)). The height increases from green to brown to white. (For interpretation of the references to color in this figure legend, the reader is referred to the web version of this article.)

According to the United Nations (United Nations, 2018), one-third of the world population will live in cities of at least 500,000 inhabitants in 2030. Those cities are facing a challenge of meeting the needs of numerous inhabitants for improving their livelihood while only limited resources are available for a sustainable development. To support those cities with geographic information, our study focuses on producing maps of urban morphology for 1692 cities whose population is greater than 300,000 according to the census of the United Nations (United Nations, 2014). The location of the 1692 cities is shown in Fig. 1.

**Fig. 2:**
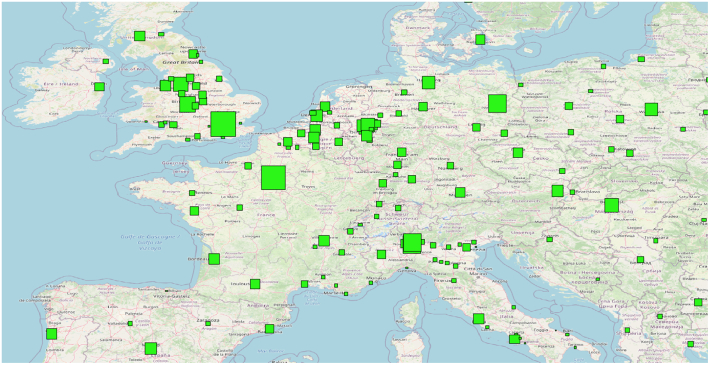
A close up of the ROIs of our cities of interest in Europe. We grow rectangles centered at the coordinates of the 1692 cities, until half of the area is not built up anymore according to GUF. Since GUF and the population figure may be out of date for certain cities, we expand the each side of the rectangles by a factor of two (i.e. factor of four in area), to take into account the rapid urbanization. Because of this extension, the ROI is much larger than the actual city boundary. As a result, some ROIs also partially overlap.

We used the existing GUF binary urban mask and the center coordinates of the 1692 cities to adaptively determine the extent of each city. We grow a rectangle centered at the coordinate of each 1692 cities, until half of the area is not built up anymore according to GUF. This serves as a basis to define the region of interest (ROI) of each city. In addition, since GUF and the population figure may be out of date for certain cities, to take into account the rapid urbanization, we expand each side of the rectangles by a factor of two (i.e. factor of four in area). In this way, we believe most built areas of cities of interest shall are included. A zoom in of the ROIs in Europe can be seen in Fig. 2. As one can see, the size of ROIs are adaptive according to the city size. Because of our extension, the ROI is much larger than the actual city boundary. As a result, some ROIs also partially overlap.

### 2.2 Data source and preprocessing

We chose the freely available Sentinel-1 and Sentinel-2 satellite data as the data sources for global urban morphology classification. These two satellite missions provide highly complementary data. Sentinel-1 provides SAR imagery in the microwave range, whereas Sentinel-2 provides multi-spectral images. Nonetheless, it is not a trivial task to extract global urban morphology from these free and open global data. The first challenge is the complex relation between the measured satellite data and the urban morphology, but also the global diversities caused by differences in cultures, environments, locations, topography, and climates. The second is the variability of the tremendous volume of satellite data for global mapping. To this end, we have developed a complete workflow of creating analysis-ready images, model training, and large-scale inferencing. Since the scope of this paper is more on the methdology and the final datasets, we briefly listed the pre-processing procedures for creating the analysis-ready data.

#### 2.2.1 Sentinel-1

Sentinel-1 level-1 single look complex (SLC) dual-pol (VV-VH) Interferometric Wide swath (IW) data of 2017 summer were collected for the 1692 cities in this study. A series of processing steps using the ESA SNAP toolbox were applied to prepare an analysis-ready dataset. These processing steps are listed as follows:

• *Apply orbit profile*: This module downloads the latest orbit profile so that a precisely geocoded product can be achieved.
• *Radiometric calibration*: This step is to provide imagery in which the pixel values can be directly related to the radar backscatter of the scene. To do this, the output scaling applied by the processor is undone and the desired scaling is reintroduced according to a look up table.
• *TOPSAR deburst*: For each polarization channel, the Sentinel-1 IW product has three swaths. Each swath image consists of a series of bursts. The TOPSAR deburst merges all these bursts and swaths into a single SLC image.
• *Polarimetric speckle reduction*: Speckle reduction was conducted by using the SNAP-integrated refined Lee filter. Unfiltered data can be preserved by skipping this module.
• *Terrain correction*: This step geocodes the range-azimuth SAR image to a geographic coordinate. SRTM DEM was used to provide the height information. The data was re-sampled to a 10m GSD by the nearest-neighbor interpolation. The data was geocoded into the WGS84/UTM coordinate system.

After processing, seven real-valued bands are contained in the Sentinel-1 analysis-ready data. They are listed in Table 1.

**Table 1:** Description of Sentinel-1 analysis-ready data

Band	Description
1	the intensity of unfiltered VH channel, in decibels
2	the intensity of unfiltered VV channel, in decibels
3	the coherence between unfiltered VV and VH
4	the intensity of the refined LEE filtered VH channel, in decibels
5	the intensity of the refined LEE filtered VV channel, in decibels
6	the coherence between the refined LEE filtered VV and VH
7	the phase difference of the refined LEE filtered VV and VH

#### 2.2.2 Sentinel-2

Similarly, Sentinel-2 L1C data (top of atmosphere reflectance) were collected for our study area. The preprocessing Sentinel-2 images is less demanding than that of Sentinel-1. The main challenge is creating cloud-free mosaics. We achieved this by exploring an engineering approach relying on pixel-wise cloud detection and the combination of multi-temporal images within short time periods (Schmitt et al., 2019). The Sentinel-2 L1C top of atmosphere reflectance data were also scaled by a factor of 1/10,000 (Gatti and Bertolini, 2013). In the Sentinel-2 analysis-ready data, 10 out of the 13 bands were used. Specifically, the channels with ground sampling distance (GSD) of 10 m and 20 m were used. The 10 real-valued bands are listed in Table 2. In addition, considering the much larger seasonal variation in optical images in comparison to SAR images, we collected a four-seasonal set of Sentinel-2 images of our study areas. The seasons collected were the winter of 2016, and the spring, summer, and autumn of 2017. It is however worth to mention that some of the cities in our study areas do not have four seasonal data due to an excessive amount of cloud or data corruption. For those cities, we only include seasons that have data available.

**Table 2:** Description of Sentinel-2 bands used in this study.

Band	Central wavelength [nm]	GSD [m]	Description
B2	490	10	Blue
B3	560	10	Green
B4	665	10	Red
B5	705	20, upsampled to 10m	Visible and Near Infrared (VNIR)
B6	740	20, upsampled to 10m	VNIR
B7	783	20, upsampled to 10m	VNIR
B8	842	10	VNIR
B8a	865	20, upsampled to 10m	VNIR
B11	1610	20, upsampled to 10m	Short Wave Infrared (SWIR)
B12	2190	20, upsampled to 10m	SWIR

### 2.3 The training dataset: So2Sat LCZ42

In order to train a deep learning model with good generalization abilities for inferencing with global data, a large-scale and representative annotated dataset is crucial. For this purpose, we created a rigorously labeled reference dataset: the *So2Sat LCZ42* benchmark dataset (Zhu et al., 2020). Over one month, 15 domain experts carefully designed the labeling workflow, the error mitigation strategy, and the validation methods, and then conducted the data labeling. The dataset consists of manually assigned LCZ labels of 400,673 Sentinel-1 and Sentinel-2 image patch pairs globally distributed in 42 urban agglomerations plus 10 additional smaller areas covering all the inhabited continents and 10 different cultural zones. We conducted a rigorous quantitative evaluation of 10 cities in the dataset by having a group of remote sensing experts cast 10 independent votes on each labeled polygon, in order to identify possible errors and assess the human labeling accuracy. The “human confusion matrices” per polygon and per pixel were created, where the confident of individual classes can be seen. In general, our human labels achieve 85% confidence. This confidence number can serve as a reference accuracy for the machine learning models trained on this dataset.

## 3 Method

### 3.1 Deep learning model

**Fig. 3:**
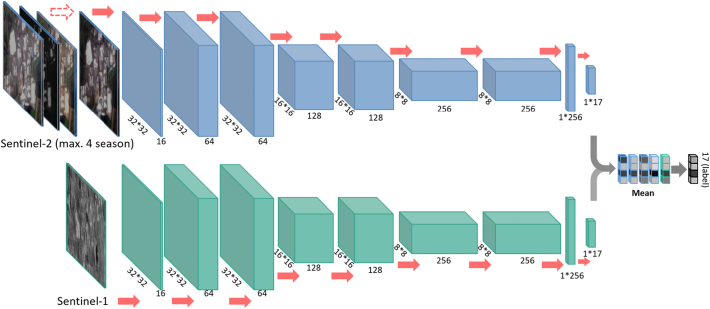
The deep learning model architecture employed in this study. This is a two streams of identical residual network with 20 layers for Sentinel-1 and Sentinel-2 image patches respectively. The Sentinel-2 stream takes input from a maximum of 4 seasonal Sentinel-2 images of a city, whereas only one season was considered for Sentinel-1. The softmax layer of all the predictions are fused via averaging, yielding the final prediction.

We employed a modified residual neural network as our base model, because multiple literatures have shown CNN greatly outperforms random forest in LCZs classification accuracy (Yoo et al., 2019, Rosentreter et al., 2020). In addition, a residual network has the advantage over many other networks in facilitating model training and encouraging feature reuse, enabled by the short-cut connections within the network. Our network consists of one convolutional layer in the beginning, six following residual blocks, and one dense layer at the end, as shown in Fig. 3. Each convolutional layer is followed by one batch normalization layer and one activation layer. Unlike the well-known ResNet 101 or 152, our modified ResNet has a reduced number of residual blocks, in order to fit the input patch size of a typical LCZ classification problem. LCZ classification maps usually have a GSD of 100 meters. We choose an input patch size of 32 × 32, which corresponds to 320 × 320 meters. This choice is a balance between the depth of the network and the definition of LCZ.

Similar to many CNNs for image classification, our model progressively reduces the resolution of the feature maps by 2. This progressive reduction of spatial resolution and increase of the number of the feature maps compress the spatial information into learned features of large dimensionalities. While optimizing with LCZ labels that emphasize the composition of land surfaces, the learned features would find a balance between the spatial correlation and the land surface composition for the classification purpose. One skip connection is applied within each residual block, which is the core characteristic of residual networks. A global average pooling layer is applied to the final 8 × 8 ×256 feature map, resulting in a 1 × 256 feature vector. This feature vector is further input to the following dense layer with a softmax activation function, outputting a 1 × 17 vector of softmax probability in the end. The softmax probability indicates the LCZ label of the input image patch. The output softmax probability is used for loss calculation together with the corresponding input reference label during the training phase.

### 3.2 Fusion of Sentinel-1 and Sentinel-2

There are two main advantages of fusing the classification results from Sentinel-1 and Sentinel-2 data. First, Sentinel-1 SAR images and Sentinel-2 multispectral images have complementary information. For example, the multi-spectral information of Sentinel-2 images provides spectral reflectance of covering materials, and the side-looking Sentinel-1 SAR data provides information of building structure and orientations. Second, Sentinel-1 data is not affected by weather and clouds. It provides more consistent classification results than Sentinel-2 data for large areas. We carried out the fusion at the decision level rather than the geometric or feature level, because of the different modality and geometry of Sentinel-1 and Sentinel-2 data. Decision level fusion averages the softmax probability of corresponding Sentinel-1 and Sentinel-2 data patches. The final prediction is the class with the highest mean probability.

### 3.3 Training strategy

In order to demonstrate a full spectrum of global performance of our deep learning model, we consider three training-testing splits: “*random-split*”, “*block-split*” and “*cultural-10*”. Among them, random-split indicates a spatially random sampling of evaluation points, which is commonly used in remote sensing. Since the distributions of training and test data are similar, random-split defines the upper bound of achievable classification accuracy. In contrast, block-split is a deterministic data split, i.e., the data from each city is separated into non-overlapping east-west blocks and the accuracy is evaluated on unseen blocks. The block-split gives a representative measure of accuracy for unseen cities whose data distribution is similar to the training cities. Last but not least, cultural-10 defines the lower bound of the achievable accuracy by evaluating completely held-out data in a cross-validation scheme. We have selected 10 held-out cities, which come from 10 different culturally-defined geographical regions of the Earth. For each city, we trained the model with all other 41 cities and then evaluated on this held-out city. Finally results of the 10 cities were averaged. In this way, “cultural-10” provides a completely unbiased view of the accuracy achievable on absolutely unseen data. Together, these three evaluation scenarios give a solid impression of the quality of the global LCZ maps produced through this approach.

### 3.4 Implementation details

The implementation of the network is under the framework of Keras with Tensorflow backend. For the training, the batch size was set to 32. We apply Adam (Kingma and Ba, 2014) as the optimizer with a learning rate of 1e-4. The number of training epochs was set to 300. Early stop was enabled with a patience of 30 epochs. The training was performed on NVIDIA Tesla P100 GPU.

## 4 Experimental results

### 4.1 Performance assessment

#### 4.1.1 General assessment

We evaluated the performance of our deep learning model based on the three data splits mentioned in the previous section. Our deep learning model shows consistent and promising accuracies in the three scenarios, which are listed in Table 3. It shows the average accuracies of using Sentinel-1 image only, average of 4 seasonal Sentinel-2 images, and the fusion of Sentinel-1 and Sentinel-2 images. Firstly we see that the three different evaluation strategies illustrate that our LCZ maps have an average accuracy between about 50% for cities that show characteristics disjunct from the training set, and more than 80% for cities that are fairly similar to the training distribution. This range of accuracies give us a realistic upper and lower bound of the performance of our model. Therefore, we are confident that the model will perform reasonably well on completely unseen cities. It should also be noted that average accuracy is a pessimistic metric compared to the more commonly used overall accuracy, as it is not weighted by the number of samples of a class. Secondly, it is obvious that 4 seasonal Sentinel-2 image (even the 1-season Sentinel-2 image, which are not listed) consistently outperform the Sentinel-1 image. The reason is not only the averaging of the results from 4 seasons, but also the successful cloud removal performed on the Sentinel-2 images. Cloud-free optical images are more suitable for vision tasks such as LCZ classification. However, the importance of Sentinel-1 image shall not be downplayed, as cloud-free Sentinel-2 images are not always available. It is estimated that 60–70% of the global Sentinel-2 images are cloudy. Therefore, our algorithm includes the fusion of Sentinel-1 and Sentinel-2 results. Table 3 shows that the fusion of SAR and optical data consistently leads to the best results. Although, the improvement is not significant, the inclusion of Sentinel-1 image will play an important role in large scale or even global scale inference, in particular for cloudy areas such as western Africa and Amazon forest.

**Table 3:** Averaged accuracy of Sentinel-1 and Sentinel-2 data, and fused results, from three types of assessment: random-split, block-split, and held-out cultural-10 split.

	Random	Block	Culture-10
Sentinel-1	64.2%	51.6%	34.0%
Sentinel-2 (4 seasons)	82.2%	75.7%	51.0%
Sentinel-1 + Sentinel-2 (4 seasons)	83.4%	76.8%	51.3%

#### 4.1.2 Per-class analysis

**Fig. 4:**
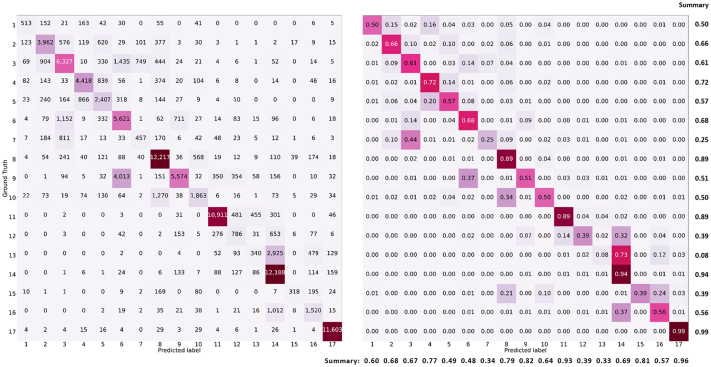
Confusion matrix of the Sentinel-1 and Sentinel-2 fusion model in the culture-10 split. Left: The value in each cell is the number of test samples. Right: the values are accuracies. A summary of the class-wise accuracy is also listed: on the right side is recall, on the bottom is precision. The classes are 1: compact high-rise, 2: compact mid-rise, 3: compact low-rise, 4: open high-rise, 5: open mid-rise, 6: open low-rise, 7: lightweight low-rise, 8: large low-rise, 9: sparsely built, 10: heavy industry, 11: dense trees, 12: scattered trees, 13: bush scrub, 14: low plants, 15: bare rock or paved, 16: bare soil sand, 17: water.

In order to get insight for future improvements, we studied the performance of our fused deep learning model in each LCZ class. The classification confusion matrix is shown in Fig. 4. We observed that certain LCZ classes are more easily confused in both Sentinel-1 and Sentinel-2 data than other classes. This can be seen both in the confusion matrices of our deep learning models as well as in the produced LCZ classification maps. Overall, we discovered three types of major confusions between the LCZ classes.

• Among the low-rise classes, including compact low-rise (class 3), large low-rise (class 6), and light-weight low-rise (class 7): About 45% lightweight low-rise (class 7) samples in our reference data were classified as compact low-rise (class 3). We also visually observe some confusion of light-weight low-rise with large low-rise in the predicted LCZ classification map of cities not in our reference data. These three classes have similar patterns of compactness. Their fundamental difference is the weight of their construction materials – information that is hard to retrieve from Sentinel-1 and Sentinel-2 images. Another reason for confusion is the limited amount of reference data in the lightweight low-rise class.
• Between heavy industry (class 10) and large low-rise (class 8): This is a reasonable confusion as large low buildings, such as factories and warehouses, are normally part of heavy industrial areas.
• Among bush, scrub (class 13), low plants (class 14), and bare soil or sand (class 16): The differences among these three classes mainly lie in the percentage of vegetation coverage, as well as on the vegetation species. Seasonal variation and the limited resolution of Sentinel-1 and Sentinel-2 data restrict their performance in discriminating above-mentioned differences.

### 4.2 Global maps of urban morphology

#### 4.2.1 Visual analysis

**Fig. 5:**
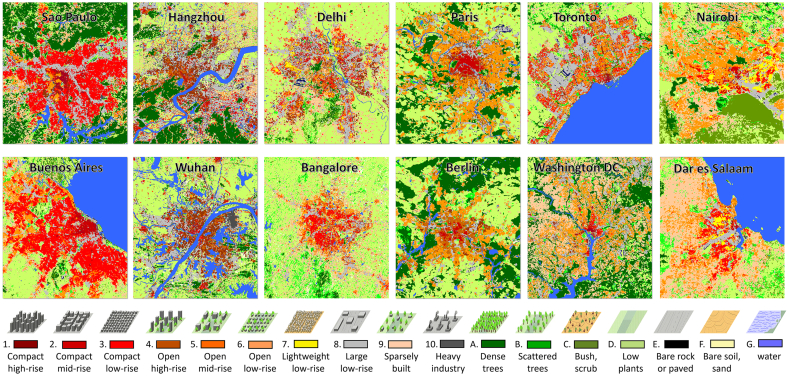
Examples of the global urban morphological maps. We employed a deep learning model on Sentinel-1 and four-seasonal Sentinel-2 data to cover the largest 1692 urban agglomerations with population greater than 300,000 according to UN's World Urbanization Prospects 2014 (United Nations, 2014). A few examples of the produced LCZ maps are sorted here according to the morphological type of the urban area. From left to right, it shows a decrease of compactness and an increase of the mixture of different LCZ classes. Note the strong correlation between the morphological type and the geographic location. A more detailed analysis of the urban morphological pattern and culture can be found in the next section. At the bottom of the figure is the legend and schematic drawings of each LCZ class. Please note that the maps of a few cities in this figure are enlarged for better visualization. They are not true in proportion.

In total, we mapped 1.65 million square kilometers of urban areas of the 1692 cities. LCZ classification maps of those cities show that our deep learning model has a strong capability of classifying urban morphology. Fig. 5 shows a few typical examples of cities with different morphological types in our study area. It can be seen that LCZ classification maps are capable of capturing different types of urban morphologic compositions, e.g., many South American cities feature large compact high-rise areas, whereas European cities feature a mixture of open mid-rise and compact mid-rise areas.

**Fig. 6:**
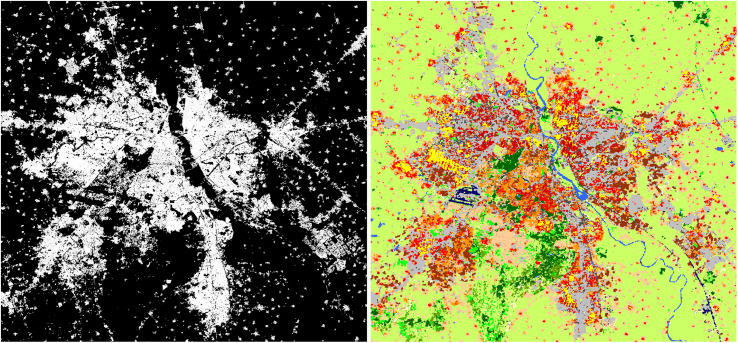
Comparison of the state-of-the-art global urban map and LCZ classification. The left image is the urban and nonurban binary classification of Delhi from the global urban footprint. The right image is the corresponding LCZ classification.

Comparing to the state of the art, i.e., binary urban-nonurban maps, our classification provides fine grained intra-urban morphological variations. Fig. 6 compares GUF (left) and our morphological map (right) of Delhi. Our morphological map makes it possible to localize the location of morphological variations within the settlement landscape, and further determine their exact morphological types. For example, the Delhi LCZ map shows there is no large compact high rise district in Delhi as we see in Sao Paulo. The dense areas is Delhi are scattered across the city. We also observe large areas of large low-rise buildings, which could be related to informal settlements. Water resources such as rivers and lakes as well as vegetation are also clearly mapped. This is important information that can aid decision making on urban development.

**Fig. 7:**
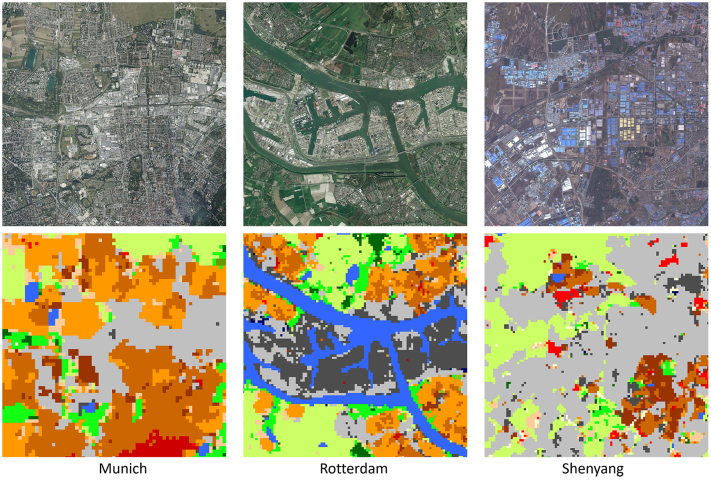
Examples of industrial areas identified in LCZ maps. From left to right, it shows the north of Munich, Germany, where the BMW factory and many historical factories are located, the port area of Rotterdam, and the industrial area of Shenyang, a typical industrial city in China. The upper image is cited from Bing Map.

Some LCZ classes also have strong correlation with certain settlement types, such as class 8 large low-rise vs. factory and warehouse, and class 7 lightweight low-risevs. informal settlements. Therefore, LCZ maps could be employed to identify those areas. In Fig. 7, we listed a few examples of industrial areas identified in the LCZ maps. It shows the north of Munich, Germany, where the BMW factory and many historical factories are located, the port area of Rotterdam, and the industrial area of Shenyang – a typical industrial city in China. These information provide a significant step in how we can perceive cities, with more accuracy, to make better planning decisions in urban areas. We make those data available to the community.

#### 4.2.2 Quantitative analysis

**Fig. 8:**
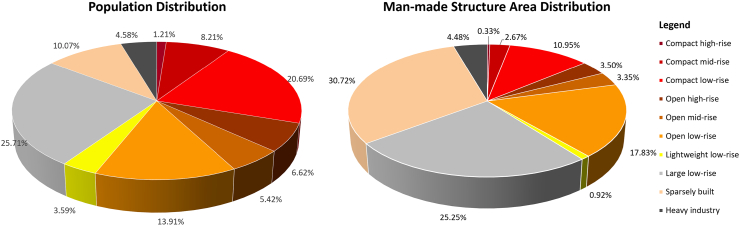
The proportions of population (left) and area (right) of the ten LCZ built up classes (classes 1–10) in the 1692 cities.

The LCZ classification maps of the largest 1692 cities provide the possibility to analyse the composition of global urban areas for the first time. In the 1.65 million km^2^ we mapped, about 550 thousand km^2^ are built-up areas, i.e. the LCZ class 1 to 10. Among these 10 classes, the sparsely built, large low-rise, open low-rise, and compact low-rise occupy the four largest proportions by area, as shown in Fig. 8 right. The largest built-up area is sparsely built, accounting for about 30% of the 1692 cities. More than 26% of the built-up area are large/lightweight low-rise buildings, often warehouses and informal human settlements. The open and compact classes occupy about another 25% and 14%, respectively, often taking the form of residential and commercial buildings.

Alarmingly, using the population of Global Human Settlement Layer in year 2015 (Schiavina et al., 2021), we found that the 30% sparsely built areas only accommodates 10% of the total population of the 1692 cities. In contrast, more than 30% of the population lives in the 14% compact areas. The difference in population density in these two types of settlement is about seven-fold at a global scale. The population proportion of each built up class can be seen in the left of Fig. 8. To see the full picture, we summarize the area and population of the four aggregated classes, i.e., compact, lightweight/large low-rise, open, and sparsely built, in Table 4. To summarize, 60% of the population live in compact and lightweight/large low-rise areas, occupying 40% of the built up area. Another 35% of the population lives in open or sparsely built areas, occupying 55% of the areas. The remaining 5% of the population is distributed in the non-built-up LCZ classes. This huge difference in population density reflects a possible inequality in living conditions.

**Table 4:** Area and population of human settlements of different compactness in the 1692 cities. From left to right, the compactness of the settlement decreases, and the population density increases.

	Compact	Lightweight / large low-rise	Open	Sparsely built
% in built up area	13.95%	26.17%	24.68%	30.72%
% of population	30.11%	29.30%	25.95%	10.07%
population per km^2^	9122	4733	4445	1385

## 5 Pilot application: empirical study of LCZ patterns vs. culture

The new availability of intra-urban LCZ classifications describing the morphologic configuration of urban landscapes enables new empirical science on cities. It enables applications in a wide variety of thematic domains, such as urban heat islands, population estimation, or analyses directly related to building and open space structures and patterns. We illustrate this capability here, with one example, by presenting a morphologic categorization of city types.

Traditionally, urban structural models have been developed in urban geography for different geographical-cultural areas, such as for the Western or the Islamic world. These models have been based on theories and observations. Our LCZ classifications now allow us to test whether groups morphologically form according to geographical-cultural regions.

To test whether spatial patterns of LCZs in cities within the same geographic-cultural spaces are more similar in their quantitative appearance as well as in their spatial composition than compared to others, we conducted the following approach: a clustering based on unsupervised methods (k-means (Hartigan and Wong, 1979) as well as expectation-maximization (Dempster et al., 1977) algorithms) is applied on the quantity and spatial location of LCZs across all cities. The optimal number of clusters is determined by the gap statistics algorithm (Tibshirani et al., 2001). Various feature spaces are tested, combining the occurrence of the 17 LCZs in general and with respect to their locations in the city; the city size is integrated as a feature as well (Taubenböck et al., 2020). We rely on comparable spatial baselines of city extents based on a consistently applied morphologic delineation method (Taubenböck et al., 2019). As it is a priori unclear which feature space and which clustering approach fits best, we evaluate our resulting clusters in relation to geographical-cultural regions based on predefined regions, using those defined by Huntington (Huntington, 1997). Using the Simpson Evenness Index (SIEI) (Simpson, 1949), we define the clusters closest as the best result.

**Fig. 9:**
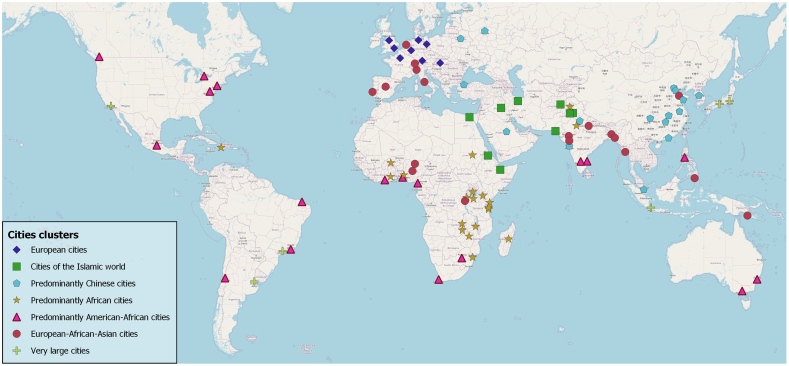
Seven resulting clusters of morphological city pattern types. Findings are based on the k-means algorithm using an 18-dimensional feature space (consisting of 17 LCZs as well as city size as features) at the spatial unit of the morphological urban areas.

We find that the resulting clusters of cities of similar morphological patterns of the built environment do reflect geographic-cultural regions to a certain degree (Fig. 9). It can be seen that Central European cities form one cluster, and Islamic cities form another one. We also identify clusters that predominantly feature Chinese and African cities. However, we also find clusters that are more spatially complex, such as clusters containing predominantly cities in American and in African as well or European as well as African and Asian cities. One last cluster consists of very large cities, which obviously represent a specific pattern. In general this confirms that certain aspects of geographic-cultural heritage have an influence on the spatial design of urban space. However, strict geographic-cultural models are not sufficient to describe urban settlement patterns: such models would negate the complexity of global interconnections in urban development.

## 6 Perspectives and discussions

The way we physically build our cities defines our life patterns, our mobility, the quality of life, the resilience of our cities, and much more. Across the globe, there are no two identical urban configurations and yet there are many groups of cities that are similar in their morphological characteristics. The measured differences in land consumption per person testify to the variability of cities worldwide and are an expression of inequality. A uniform and consistent classification is the basis for such new and better understandings of the status quo and thus also for improving urban planning in the future.

In times of global urbanization, it must therefore be of central interest to create such a data set for cross-disciplinary use. The global high resolution and consistent local climate zone classification map generated in this manner can be the basis for various applications: just a few examples include understanding the urban thermal environment to mitigate urban heat islands in human settlements, assessing population distributions, and simply analyzing urban morphological configurations. Our geostatistical result exemplifies the latter, demonstrating that patterns of urban morphology have a strong correlation to the geographical region and the related cultural heritage. However, we believe that extensive urban-related researches based on information extracted from this dataset can help stakeholders to develop appropriate policies and distribute resources more effectively. Of course, we are fully aware that the LCZ scheme is only one possible conceptual approach among many others to a systematic intra-urban description of urban morphology. To what extent these 17 LCZ classes can cover the urban landscape comprehensively and also in its manifestations worldwide must be investigated in future studies. However, we believe the LCZ scheme as a generic, culturally-neutral description of land-use and land-cover (Stewart and Oke, 2012) relying on universal, standardized and measurable parameters of urban form allows for a consistent and systematic description of the built urban landscape.

Although the overall accuracy of the classification results we achieved in this work is high, the discrepancy of accuracy between different classes, particularly, height-related classes, is obvious. The complementary Sentinel-1 data can improve the result. However, the moderate resolution of Sentinel-1 limited significant improvement. We have investigated the potential of using TanDEM-X data, whose spatial resolution is much higher than Sentinel-1 data, for the same task. Our preliminary experiments show promising results, suggesting that higher resolution SAR data would significantly complement the lacking height information. Since the TanDEM-X bistatic data provides global coverage and is free from clouds, a higher resolution and better quality classification dataset could potentially be achieved in the future to enable more precise analysis in supporting different applications.

In general, the volume of open-access remote sensing data is increasing significantly. The proposed deep learning based multi-sensor fusion framework exemplifies the potential of developing advanced machine learning pipelines for extracting desired information from such a “gold mine.” Together with high performance computing (HPC), high-quality geo-information layers that currently do not exist can be efficiently generated at a global scale from peta bytes of EO data. This offers a great perspective toward filling the geo-information gap between knowledge and strategic directions, e.g., while addressing United Nations Sustainable Development Goals, or monitoring mega trends such as climate change and global urbanization.

## Author contributions

Conceptualization, X.Z.; methodology, X.Z., C.Q., J.H., Y.S., Y.W., M.S., H.T.; software, C.Q., J.H., Y.S., Y.W.; results validation, C.Q., J.H.; investigation, H.T., X.Z., J.H.; data curation: C.Q., J.H., Y.S., M.S.; writing - original draft, all authors; writing - review & editing: X.Z, Y.W., H.T., J.H., M.S., Y.S.; visualization, J.H., Y.W., H.T.; funding acquisition, X.Z.

## Funding

The work is jointly supported by the European Research Council (ERC) under the European Union's Horizon 2020 research and innovation programme (grant agreement No. [ERC-2016-StG-714087], Acronym: *So2Sat* and grant agreement No. [957407], Acronym: *DAPHNE*, by the Helmholtz Association through the Framework of Helmholtz AI (grant number: ZT-I-PF-5-01) - Local Unit “Munich Unit @Aeronautics, Space and Transport (MASTr)”, the Helmholtz Klimainitiative (HICAM) and Helmholtz Excellent Professorship “Data Science in Earth Observation - Big Data Fusion for Urban Research” (grant number: W2-W3-100)) and by the German Federal Ministry of Education and Research (BMBF) in the framework of the international future AI lab “AI4EO – Artificial Intelligence for Earth Observation: Reasoning, Uncertainties, Ethics and Beyond” (grant number: 01DD20001).

## Declaration of Competing Interest

Xiaoxiang Zhu reports financial support was provided by European Research Council. Xiaoxiang Zhu reports financial support was provided by the Helmholtz Association of German Research Centers eV. Xiaoxiang Zhu reports financial support was provided by German Federal Ministry of Education and Research.

## Footnotes

2 data: https://doi.org/10.14459/2021mp1633461, demo: https://github.com/zhu-xlab/So2Sat-LCZ-Classification-Demo
